# Supplementary material for: Comprehensive Assessment of Indian Variations in the Druggable Kinome Landscape Highlights Distinct Insights at the Sequence, Structure and Pharmacogenomic Stratum
Source: Front Pharmacol. 2022 Jul 5;13:858345. doi: 10.3389/fphar.2022.858345 (PMC9294532; doi:10.3389/fphar.2022.858345)
Supplement: Supplementary file 3 [file DataSheet1.pdf]

SUPPLEMENTARY MATERIAL

Comprehensive assessment of Indian variations in the druggable kinome landscape highlights distinct insights at the sequence, structure and pharmacogenomic stratum.

Gayatri Panda1‡, Neha Mishra1‡, Disha Sharma2,3, Rintu Kutum 2,3 Rahul C. Bhoyar3, Abhinav Jain2,3, Mohamed Imran2,3, Vigneshwar Senthilvel2,3, Mohit Kumar Divakar2,3, Anushree Mishra3, Parth Garg1, Priyanka Banerjee4, Sridhar Sivasubbu2,3, Vinod Scaria2,3, Arjun Ray1*

1 Department of Computational Biology, Indraprastha Institute of Information Technology, Okhla, India.
2 Academy of Scientific and Innovative Research (AcSIR), Ghaziabad, India.
3 CSIR-Institute of Genomics and Integrative Biology, Mathura Road, Delhi-110020, India.
4 Institute for Physiology, Charit_e-University Medicine Berlin, 10115 Berlin, Germany.
‡These authors contributed equally to this work.
* arjun@iiitd.ac.in

1 SUPPLEMENTARY FIGURES

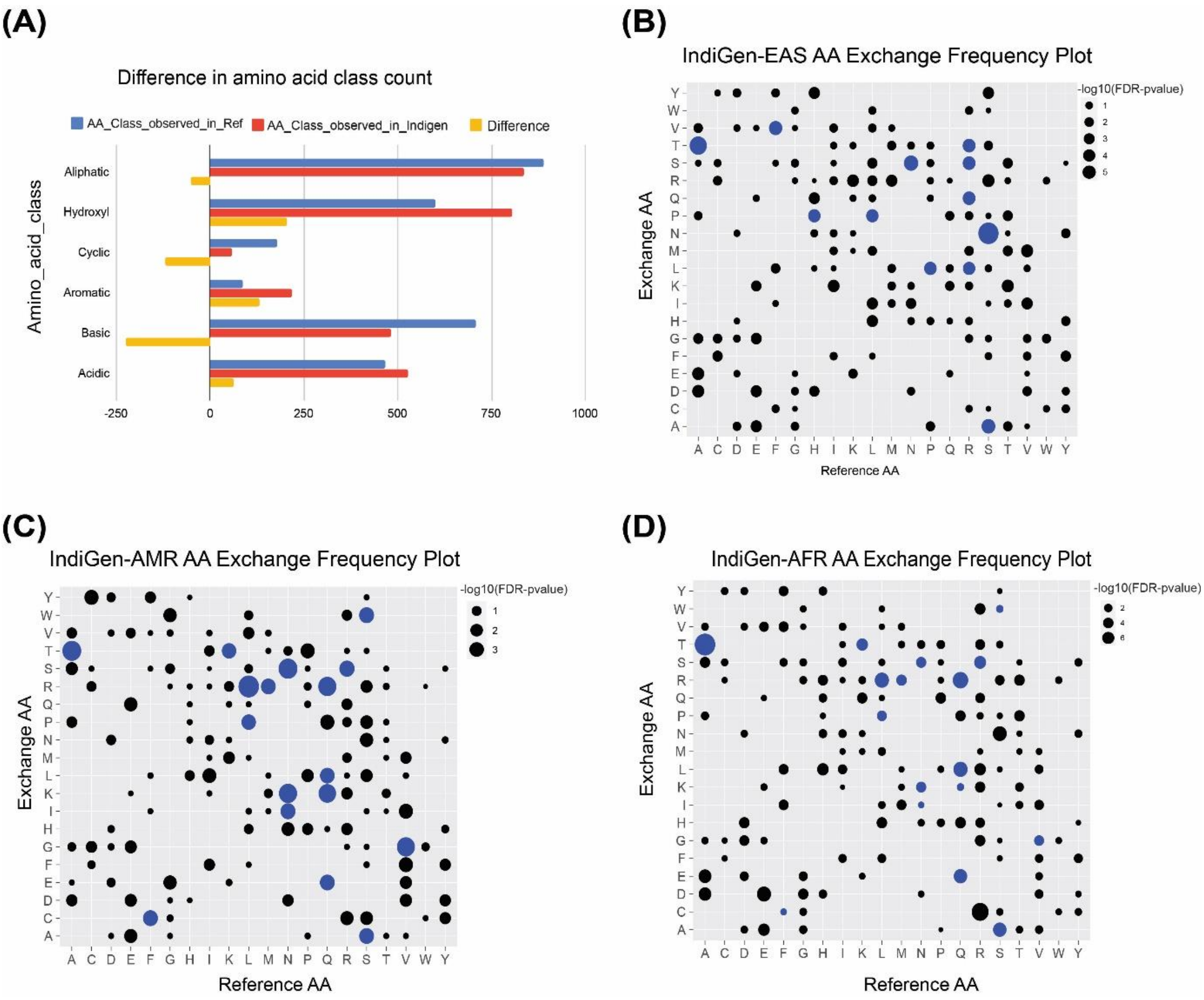

Figure_S1: ***A***. *Chemical changes observed from reference amino acid (RefSeq (hg38)) to alternative amino-acids at SNP sites reported in IndiGen data.* ***B,C,D***. *Bubble-plot was generated on the basis of*

the FDR corrected p-value associated with AA-exchange frequency for a particular Reference and Alternative AA observed in IndiGen data with EAS , AMR and AFR populations of 1000 genome data.

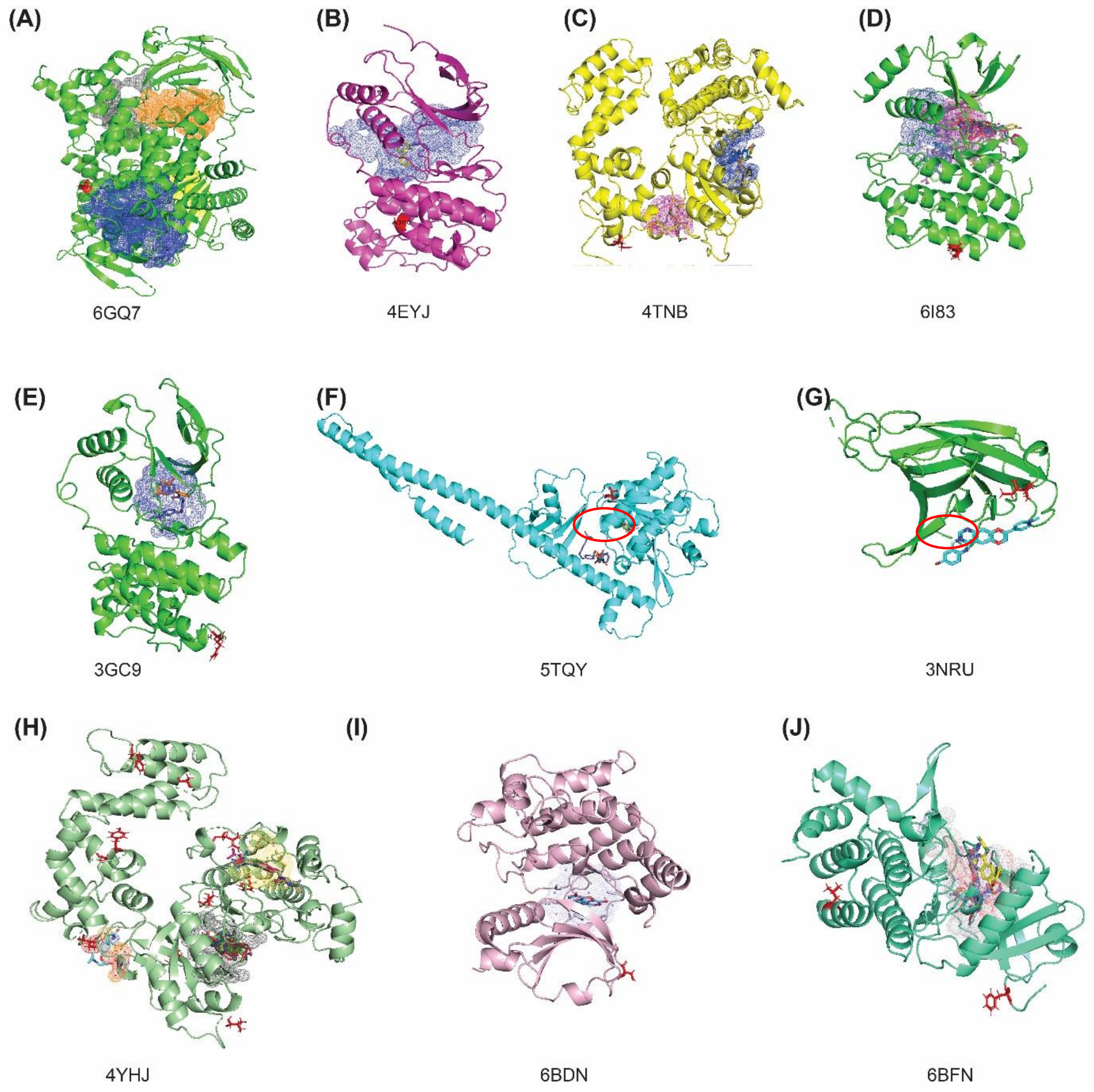

Figure_S2. (**A-G)**. *Snapshot of docked complexes* ***A.*** *6GQ7 docked with 34 ligands bound at 4 different pockets (grey, blue, orange and yellow color), mutated residue Thr at 857th position shown in red stick representation.* ***B.*** *4EYJ docked to 1 ligand (blue pocket).* ***C.*** *4TNB docked with 4 ligands in two pockets (pink, blue color).* ***D.*** *6I83 docked with 15 ligand molecules in two pockets (purple and pink).* ***E.*** *3GC9 docked with 2 ligands in one pocket(blue).* ***F.*** *5TQY docked with 5 ligands in one pocket (red circle).* ***G****. 3NRU docked with 1 ligand in one pocket (red circle)* ***H.*** *4YHJ docked with 4 ligands in 3pockets (grey, orange, yellow).* ***I.*** *6BDN docked with 1 ligand a pocket (purple)* ***J.*** *6BFN docked with 2 ligands in 1 pocket (orange)*

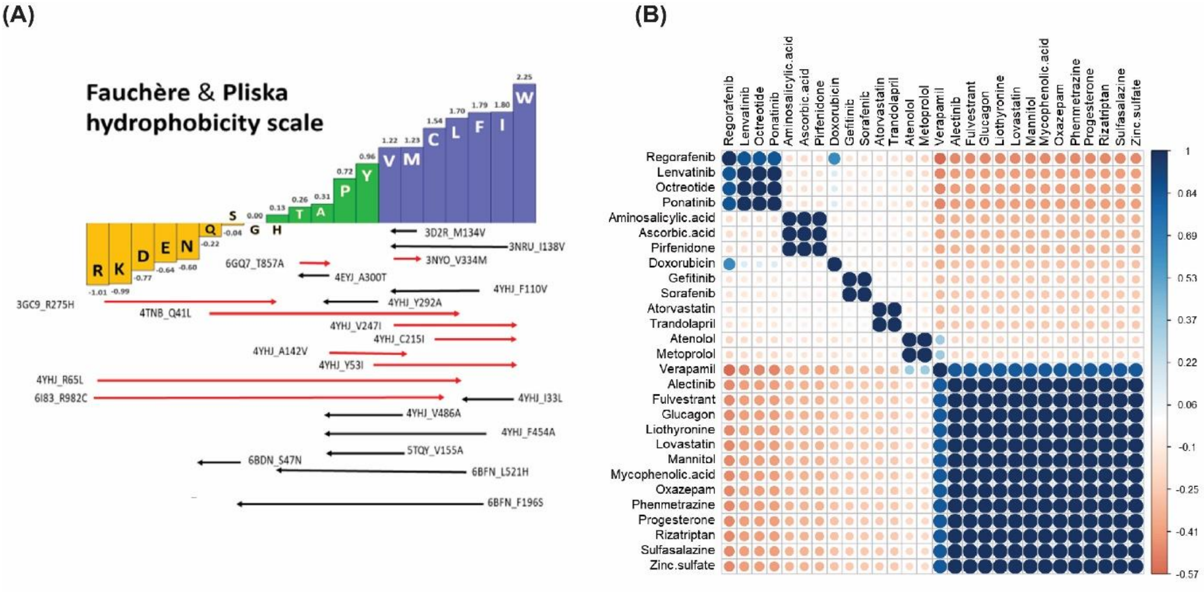

Figure_S3. ***A***. *Fauchere and Pliska hydrophobicity scale showing change in hydrophobicity observed in 22 mutations (red-line for increase in hydrophobicity and black line for decrease in hydrophobicity).* ***B.*** *Phenotypic drug-drug correlogram*

2 SUPPLEMENTARY TABLES

Table_S1: 545 druggable kinase coding genes for the study.

AAK1	CDK13	EPHA4	LATS2	MKNK1	PIK3R5	ROCK2	TEC
AATK	CDK14	EPHA5	LCK	MKNK2	PIK3R6	ROR1	TEK
ABL1	CDK15	EPHA6	LIMK1	MLKL	PIM1	ROR2	TESK1
ABL2	CDK16	EPHA7	LIMK2	MOK	PIM2	ROS1	TESK2
ACVR1	CDK17	EPHA8	LMTK2	MOS	PIM3	RPS6KA1	TEX14
ACVR1B	CDK18	EPHB1	LMTK3	MST1R	PINK1	RPS6KA2	TGFBR1
ACVR1C	CDK19	EPHB2	LRRK1	MTOR	PIP4K2A	RPS6KA3	TGFBR2
ACVR2A	CDK2	EPHB3	LRRK2	MUSK	PIP4K2B	RPS6KA4	TGFBR3
ACVR2B	CDK20	EPHB4	LTK	MYLK	PIP4K2C	RPS6KA5	TIE1
ACVRL1	CDK3	EPHB6	LYN	MYLK2	PIP5K1A	RPS6KA6	TLK1
ADCK1	CDK4	ERBB2	MAK	MYLK3	PIP5K1B	RPS6KB1	TLK2
ADCK2	CDK5	ERBB3	MAP2K1	MYLK4	PIP5K1C	RPS6KB2	TNIK
ADCK5	CDK6	ERBB4	MAP2K2	MYO3A	PKDCC	RPS6KC1	TNK1
AKT1	CDK7	ERN1	MAP2K3	MYO3B	PKMYT1	RPS6KL1	TNK2
AKT2	CDK8	ERN2	MAP2K4	NEK1	PKN1	RYK	TNNI3K
AKT3	CDK9	FER	MAP2K5	NEK10	PKN2	SBK1	TP53RK
ALK	CDKL1	FES	MAP2K6	NEK11	PKN3	SBK2	TRIB1
ALPK1	CDKL2	FGFR1	MAP2K7	NEK2	PLK1	SBK3	TRIB2
ALPK3	CDKL3	FGFR2	MAP3K1	NEK3	PLK2	SCYL1	TRIB3
AMHR2	CDKL4	FGFR3	MAP3K10	NEK4	PLK3	SCYL2	TRIO
ANKK1	CDKL5	FGFR4	MAP3K11	NEK5	PLK4	SGK1	TRPM6
ARAF	CERK	FGR	MAP3K12	NEK6	PNCK	SGK2	TRPM7
ATM	CHEK1	FLT1	MAP3K13	NEK7	PRKAA1	SGK3	TSSK1B

ATR	CHEK2	FLT3	MAP3K14	NEK8	PRKAA2	SIK1	TSSK2
AURKA	CHUK	FLT4	MAP3K15	NEK9	PRKAB1	SIK2	TSSK3
AURKB	CIT	FOXN3	MAP3K19	NIM1K	PRKAB2	SIK3	TSSK4
AURKC	CLK1	FRK	MAP3K2	NLK	PRKACA	SLK	TSSK6
AXL	CLK2	FYN	MAP3K20	NRBP1	PRKACB	SMG1	TTBK1
BCKDK	CLK3	GAK	MAP3K3	NRBP2	PRKACG	SNRK	TTBK2
BCR	CLK4	GCK	MAP3K4	NRK	PRKAG1	SPEG	TTK
BLK	COQ8A	GRK1	MAP3K5	NTRK1	PRKAG2	SPHK1	TTN
BMP2K	CSF1R	GRK4	MAP3K6	NTRK2	PRKAG3	SPHK2	TWF1
BMPR1A	CSK	GRK5	MAP3K7	NTRK3	PRKAR1A	SRC	TWF2
BMPR1B	CSNK1A1	GRK6	MAP3K8	NUAK1	PRKAR1B	SRMS	TXK
BMPR2	CSNK1A1L	GRK7	MAP3K9	NUAK2	PRKAR2A	SRPK1	TYK2
BMX	CSNK1D	GSK3A	MAP4K1	OBSCN	PRKAR2B	SRPK2	TYRO3
BRAF	CSNK1E	GSK3B	MAP4K2	OXSR1	PRKCA	SRPK3	UHMK1
BRD1	CSNK1G1	GUCY2C	MAP4K3	PAK1	PRKCB	STK10	ULK1
BRD2	CSNK1G2	GUCY2D	MAP4K4	PAK2	PRKCD	STK11	ULK2
BRD3	CSNK1G3	HASPIN	MAP4K5	PAK3	PRKCE	STK16	ULK3
BRD4	CSNK2A1	HCK	MAPK1	PAK4	PRKCG	STK17A	ULK4
BRD7	CSNK2A2	HIPK1	MAPK10	PAK5	PRKCH	STK17B	VRK1
BRD8	CSNK2B	HIPK2	MAPK11	PAK6	PRKCI	STK19	VRK2
BRD9	CSRP2	HIPK3	MAPK12	PASK	PRKCQ	STK24	VRK3
BRSK1	CSRP2P1	HIPK4	MAPK13	PBK	PRKCZ	STK25	WEE1
BRSK2	DAPK1	HUNK	MAPK14	PDGFRA	PRKD1	STK26	WEE2
BTK	DAPK2	ICK	MAPK15	PDGFRB	PRKD2	STK3	WNK1
BUB1	DAPK3	IGF1R	MAPK3	PDIK1L	PRKD3	STK31	WNK2
BUB1B	DCLK1	IKBKB	MAPK4	PDK2	PRKDC	STK32A	WNK3
CAMK1	DCLK2	IKBKE	MAPK6	PDK3	PRKG1	STK32B	WNK4
CAMK1D	DCLK3	ILK	MAPK7	PDK4	PRKG2	STK32C	YES1
CAMK1G	DDR1	INSR	MAPK8	PDPK1	PRKX	STK33	ZAP70
CAMK2A	DDR2	INSRR	MAPK9	PHKG1	PRPF4B	STK35	
CAMK2B	DMPK	IRAK1	MAPKAPK2	PHKG2	PSKH1	STK36	
CAMK2D	DSTYK	IRAK2	MAPKAPK3	PI4K2A	PSKH2	STK38	
CAMK2G	DYRK1A	IRAK3	MAPKAPK5	PI4K2B	PTK2	STK39	
CAMK4	DYRK1B	IRAK4	MARK1	PI4KA	PTK2B	STK4	
CAMKK1	DYRK2	ITK	MARK2	PI4KB	PTK6	STK40	
CAMKK2	DYRK3	ITPKA	MARK3	PIK3C2A	PTK7	STKLD1	
CAMKV	DYRK4	ITPKB	MARK4	PIK3C2B	PXK	STRADA	
CASK	EEF2K	ITPKC	MAST1	PIK3C2G	RAF1	STRADB	
CDC42BPA	EGFR	JAK1	MAST2	PIK3C3	RET	STYK1	
CDC42BPB	EIF2AK1	JAK2	MAST3	PIK3CA	RIOK1	SYK	

CDC42BPG	EIF2AK2	JAK3	MAST4	PIK3CB	RIOK2	TAF1	
CDC7	EIF2AK3	KALRN	MASTL	PIK3CD	RIOK3	TAF1L	
CDK1	EIF2AK4	KDR	MATK	PIK3CG	RIPK1	TAOK1	
CDK10	EPHA1	KIT	MELK	PIK3R1	RIPK2	TAOK2	
CDK11A	EPHA10	KSR1	MERTK	PIK3R2	RIPK3	TAOK3	
CDK11B	EPHA2	KSR2	MET	PIK3R3	RIPK4	TBCK	
CDK12	EPHA3	LATS1	MINK1	PIK3R4	ROCK1	TBK1	

Table_S2: Allele Frequency of variations observed in IndiGen data vs other databases.

Gene	Variation	Indigen frequency	1000 genome database	Genome AD Exome All	ExAC database	avsnp
CHUK	V155A	0.06	0.009	0.002	0.089	rs2278107
EPHA7	I138V	0.058	0.622	0.058	0.421	rs2960306
GRK4	F110V	0.196	0.136	0.3833	0.4016	rs1024323
GRK4	A142V	0.196	0.136	0.3833	0.4016	rs1024323
GRK4	C215I	0.027	0.021	0.011	0.0068	rs1140085
GRK4	Y53I	0.027	0.001	0.027	0.91	rs1140085
GRK4	Y292A	0.724	.	.	0.487	rs150897108
GRK4	V247I	0.027	0.126	0.03	.	rs1801058
GRK4	V486A	0.724	0.003	0.002	0.812	rs1801058
GRK4	F454A	0.724	0.017	0.621	0.329	rs2230345
GRK4	R65L	0.171	0.116	0.101	0.121	rs2960306
GRK4	I33L	0.171	0.063	0.106	0.212	rs2960306
GRK5	Q41L	0.074	0.002	0.003	0.014	rs2230349
GRK6	V334M	0.012	0.313	0.724	0.373	rs143935970
IRAK1	R521H	0.378	0.546	0.036	0.693	rs1059702
IRAK1	F196S	0.026	0.026	0.001	0.11	rs33932986
MAPK11	R275H	0.01	0.01	0.072	0.101	rs41270090
MAPK13	A300T	0.011	0.373	0.009	.	rs144262262
PDK4	M134V	0.011	0.106	0.043	0.201	rs17847825
PIK3CG	T857A	0.089	0.101	0.099	0.07	rs28763991
RET	R982C	0.036	0.693	0.514	0.901	rs17158558
TAOK3	S47N	0.731	0.731	0.621	0.421	rs428073

Table_S3: Allele frequency Indian v/s other populations from 1000 genome data(1000g2015).

Gene	avsnp150	AFR	EUR	SAS	EAS	AMR	Indigen
CHUK	rs2230803	0.001	0	0.048	0.055	0.001	0.06
EPHA7	rs2960306	0.537	0.378	0.155	0.094	0.329	0.171
GRK4	rs1024323	0.634	0.402	0.174	0.19	0.383	0.196
GRK4	rs1801058	0.91	0.57	0.728	0.534	0.643	0.724
GRK4	rs2960306	0.537	0.378	0.155	0.094	0.329	0.171
GRK5	rs2230349	0.002	0.076	0.205	0.283	0.095	0.074
GRK6	rs143935970	0	0	0.012	0	0.006	0.012
IRAK1	rs1059702	0.967	0.853	0.393	0.22	0.574	0.411
IRAK1	rs33932986	0.015	0.022	0.018	0	0.033	0.01
MAPK11	rs41270090	0	0	0.006	0	0.003	0.175
MAPK13	rs144262262	0	0.011	0.026	0	0.01	0.011
PDK4	rs17847825	0.007	0.11	0.239	0.193	0.048	0.216
PIK3CG	rs28763991	0.077	0.038	0.088	0.032	0.084	0.089
RET	rs17158558	0.011	0.016	0.033	0.032	0.023	0.036
TAOK3	rs428073	0.745	0.683	0.759	0.714	0.761	0.731

Table_S4: Data used for structural analysis (gene names, PDB ids and observed mutations in IndiGen data and no. of FDA-approved drugs given by DGIdb for these genes, Sequence/structure coverage)

Gene	PDB ID	Variation	#Drugs (FDA approved)	Coverage (%)
CHUK	5TQY	V155A	5	87.25
EPHA7	3NRU	I138V	1	17.23
GRK4	4YHJ	F110V	3	99.82
		Y292A		
		V247I		
		C215I		
		A142V		
		Y53I		
		R65L		
		I33L		
		V486A		
		F454A		
GRK5	4TNB	Q41L	4	99.83
GRK6	3NYO	V334M	No interaction with FDA-approved Drug	99.65
IRAK1	6BFN	L521H	3	47.19
		F196S		
MAPK11	3GC9	R275H	2	99.72
MAPK13	4EYJ	A300T	1	96.15
PDK4	3D2R	M134V	No interaction with FDA-approved Drug	95.13
PIK3CG	6GQ7	T857A	34	85.93
RET	6I83	R982C	15	27.64
TAOK3	6BDN	S47N	1	35.07

Table_S5: Data used for docking

Native	Variation	DrugBank Id
3GC9	R275H	DB04951
3GC9	R275H	DB08896
3NRU	I138V	DB05294
4EYJ	A300T	DB04951
4TNB	Q41L	DB00519
4TNB	Q41L	DB00661
4TNB	Q41L	DB00999
4TNB	Q41L	DB00335
5TQY	V155A	DB00795
5TQY	V155A	DB00244
5TQY	V155A	DB00126
5TQY	V155A	DB00233
5TQY	V155A	DB06151
6GQ7	T857A	DB00091
6GQ7	T857A	DB00104
6GQ7	T857A	DB00227
6GQ7	T857A	DB00279
6GQ7	T857A	DB00363
6GQ7	T857A	DB00388
6GQ7	T857A	DB00396
6GQ7	T857A	DB00481
6GQ7	T857A	DB00641
6GQ7	T857A	DB00655
6GQ7	T857A	DB00742
6GQ7	T857A	DB00830
6GQ7	T857A	DB00842
6GQ7	T857A	DB00947
6GQ7	T857A	DB00953
6GQ7	T857A	DB00984
6GQ7	T857A	DB00997
6GQ7	T857A	DB01024
6GQ7	T857A	DB01064
6GQ7	T857A	DB01065
6GQ7	T857A	DB01076
6GQ7	T857A	DB01152
6GQ7	T857A	DB01197
6GQ7	T857A	DB01229
6GQ7	T857A	DB01392
6GQ7	T857A	DB01394
6GQ7	T857A	DB09054
6GQ7	T857A	DB09322
6GQ7	T857A	DB11091
6GQ7	T857A	glucagon
6GQ7	T857A	neomycin

6GQ7	T857A	thyrotropin_releasing_factor
6I83	R982C	alectinin_hcl
6I83	R982C	DB00398
6I83	R982C	DB00619
6I83	R982C	DB00755
6I83	R982C	DB01234
6I83	R982C	DB01268
6I83	R982C	DB01590
6I83	R982C	DB05294
6I83	R982C	DB08875
6I83	R982C	DB08896
6I83	R982C	DB08901
6I83	R982C	DB09078
6I83	R982C	DB09079
6I83	R982C	DB11363
6I83	R982C	sorafenib_tosylate
6I83	R982C	sunitinib_malate
4YHJ	F110V	DB00264
4YHJ	F110V	DB00661
4YHJ	F110V	DB00335
4YHJ	Y292A	DB00264
4YHJ	Y292A	DB00661
4YHJ	Y292A	DB00335
4YHJ	V247I	DB00264
4YHJ	V247I	DB00661
4YHJ	V247I	DB00335
4YHJ	C215I	DB00264
4YHJ	C215I	DB00661
4YHJ	C215I	DB00335
4YHJ	A142V	DB00264
4YHJ	A142V	DB00661
4YHJ	A142V	DB00335
4YHJ	Y53I	DB00264
4YHJ	Y53I	DB00661
4YHJ	Y53I	DB00335
4YHJ	R65L	DB00264
4YHJ	R65L	DB00661
4YHJ	R65L	DB00335
4YHJ	I33L	DB00264
4YHJ	I33L	DB00661
4YHJ	I33L	DB00335
4YHJ	V486A	DB00264
4YHJ	V486A	DB00661
4YHJ	V486A	DB00335
4YHJ	F454A	DB00264
4YHJ	F454A	DB00661

4YHJ	F454A	DB00335
6BDN	S47N	DB00295
6BFN	L521H	DB00317
6BFN	L521H	DB00619
6BFN	L521H	DB00398
6BFN	F196S	DB00317
6BFN	F196S	DB00619
6BFN	F196S	DB00398

Table_S6: Data used for ligand similarity analysis

Native	Drug Name	Variation	DrugBank Id	Delta(native-mutant)
6GQ7	Zinc sulfate	T857A	DB09322	-9.1
6GQ7	Fulvestrant	T857A	DB00947	-0.3
5TQY	Pirfenidone	V155A	DB00795	-0.2
6GQ7	Verapamil	T857A	DB00997	-0.2
6GQ7	Sulfasalazine	T857A	DB01076	-0.2
3GC9	Doxorubicin	R275H	DB04951	-0.2
4TNB	Atorvastatin	Q41L	DB00661	-0.2
6GQ7	Mannitol	T857A	DB00742	-0.2
6GQ7	Rizatriptan	T857A	DB00953	-0.2
6GQ7	Mycophenolic acid	T857A	DB01024	-0.2
3GC9	Regorafenib	R275H	DB08896	-0.1
4TNB	Trandolapril	Q41L	DB00519	-0.1
5TQY	Ascorbic acid	V155A	DB00126	-0.1
5TQY	Aminosalicylic acid	V155A	DB00233	-0.1
6GQ7	Lovastatin	T857A	DB00227	-0.1
6GQ7	Liothyronine	T857A	DB00279	-0.1
6GQ7	Progesterone	T857A	DB00396	-0.1
6GQ7	Phenmetrazine	T857A	DB00830	-0.1
6GQ7	Oxazepam	T857A	DB00842	-0.1
6GQ7	Glucagon	T857A	glucagon	-0.1
6I83	Octreotide	R982C	DB09078	0.1
6I83	Lenvatinib	R982C	DB11363	0.1
6GQ7	Alectinib	T857A	DB00104	0.1
6I83	Ponatinib	R982C	DB08901	0.1
6I83	Regorafenib	R982C	DB08896	0.7
6BFN	Sorafenib	F196S	DB00398	-0.2
4YHJ	Verapamil	F110V	DB00661	-0.2
4YHJ	Atenolol	Y292A	DB00335	-0.2
4YHJ	Atenolol	A142V	DB00335	-0.1
4YHJ	Atenolol	F454A	DB00335	-0.1
4YHJ	Atenolol	V247I	DB00335	-0.1
4YHJ	Metoprolol	F454A	DB00264	-0.1
4YHJ	Verapamil	F454A	DB00661	-0.1
4YHJ	Metoprolol	V247I	DB00264	-0.1
4YHJ	Verapamil	V247I	DB00661	-0.1
4YHJ	Metoprolol	Y292A	DB00264	-0.1

4YHJ	Verapamil	Y53I	DB00661	-0.1
4YHJ	Verapamil	C215I	DB00661	0.1
4YHJ	Metoprolol	I33L	DB00264	0.1
4YHJ	Atenolol	R65L	DB00335	0.1
4YHJ	Verapamil	R65L	DB00661	0.1
4YHJ	Metoprolol	Y53I	DB00264	0.1
4YHJ	Atenolol	I33L	DB00335	0.3
6BFN	Gefitinib	L521H	DB00317	0.4
6BFN	Gefitinib	F196S	DB00317	0.5

Table_S7: **Computational prediction of toxicity profiles of drugs using ProTox-II platform**

DrugBank Ids	Drug Names	Acute Toxicity (mg/kg)	Organ Toxicity	Toxicity endpoints	Adverse outcomes pathways (AOPs)	SIDER ADR links
DB00947	Fulvestrant	2000	NA	immunotoxic (0.99)	NR_Aromatase (1.00) SR-MMP (0.98)	http://sideeffects.embl.de/drugs/104741/
DB00795	Sulfasalazine	1998	hepatotoxic (0.69)	immunotoxic (0.98)	NR-Aromatase (1.00) NR-ER (0.99) NR-ER-LBD (1.00)	http://sideeffects.embl.de/drugs/5353980/
DB00997	Doxorubicin	205	NA	immunotoxic (0.99) mutagenic (0.98) cytotoxic (0.94)	NR_Aromatase (0.52) SR_p53 (0.52)	http://sideeffects.embl.de/drugs/1690/
DB01076	Atorvastatin	5000	hepatotoxic (0.76)	NA	NR-Aromatase (1.00)	http://sideeffects.embl.de/drugs/2250/
DB04951	Pirfenidone	580	hepatotoxic (0.57)	carcinogenic (0.54)	NA	http://sideeffects.embl.de/drugs/40632/
DB00661	Verapamil	108	NA	immunotoxic (0.97)	NA	http://sideeffects.embl.de/drugs/2520/
DB00742	Mannitol	13500	NA	NA	NA	http://sideeffects.embl.de/drugs/453/
DB00953	Rizatriptan	100	NA	immunotoxic (0.69)	NA	http://sideeffects.embl.de/drugs/5078/
DB01024	Mycophenolic acid	800	hepatotoxic (0.82)	immunotoxic (0.99) cytotoxic (0.77)	SR-MMP (0.79)	http://sideeffects.embl.de/drugs/4272/
DB08896	Regorafenib	800	hepatotoxic (0.82)	immunotoxic (0.99) cytotoxic (0.77)	SR-MMP (0.79) SR-p53(0-57)	http://sideeffects.embl.de/drugs/11167602/

DB00519	Trandolapril	1800	NA	NA	NA	http://sideeffects.embl.de/drugs/5525/
DB00126	Ascorbic acid	3367	NA	NA	NA	http://sideeffects.embl.de/drugs/11020241/
DB00233	Aminosalicylic acid	4000	hepatotoxic (0.82)	NA	NA	http://sideeffects.embl.de/drugs/4649/
DB00227	Lovastatin	1000	NA	carcinogenic (0.80) immunotoxic (0.99)	NR-Aromatase (1.00) SR-MMP (0.88)	http://sideeffects.embl.de/drugs/3962/
DB00279	Liothyronine	10000	NA	NA	NR-AhR (0.94) NR-ER (0.78) NR-PPAR-Gamma (0.94) SR-MMP (0.75)	http://sideeffects.embl.de/drugs/861/
DB00396	Progesterone	2450	NA	carcinogenic (0.56) immunotoxic (0.95)	NR-AR (0.97) NR-LBD (0.97) NR-ER (0.85) SR-ARE (0.86) SR-p53 (0.80) SR-HSE (0.86)	http://sideeffects.embl.de/drugs/4920/
DB00830	Phenmetrazine	125	NA	NA	NA	
DB00842	Oxazepam	370	NA	carcinogenic (0.74)	NR-AR (0.95)	http://sideeffects.embl.de/drugs/4616/
DB09078	Lenvatinib	3000	NA	immunotoxic (0.98)	NA	
DB11363	Alectinib	2400	NA	immunotoxic (0.98)	NA	
DB00104	Octreotide	1000	NA	immunotoxic (0.74)	NA	http://sideeffects.embl.de/drugs/54373/
DB08901	Ponatinib	1190	NA	immunotoxic (0.96)	NR-Aromatase (1.00) NR-ER (0.99) NR-ER-LBD (1.00)	http://sideeffects.embl.de/drugs/24826799/
DB00317	Gefitinib	2935	hepatotoxic (0.73)	immunotoxic (0.99)	NR-AhR (1.00)	http://sideeffects.embl.de/drugs/123631/
DB00040	Glucagon	73	NA	NA	NA	NA
DB00264	Metoprolol	1050	NA	NA	NA	http://sideeffects.embl.de/drugs/4171/
DB00398	Sorafenib	800	hepatotoxic (0.82)	immunotoxic (0.92) cytotoxic (0.77)	NA	http://sideeffects.embl.de/drugs/216239/
DB09322	Zinc sulfate	448	NA	carcinogenic (0.64)	NA	http://sideeffects.embl.de/drugs/24424/

DB00335	Atenolol	2000	NA	NA	NA	http://sideeffects.embl.de/drugs/2249/

* aryl hydrogen receptor (AhR), androgen receptor (AR), androgen receptor ligand-binding domain (AR-LBD), aromatase, estrogen receptor alpha (ER), estrogen receptor ligand-binding domain (ER-LBD), and peroxisome proliferator-activated receptor gamma (PPAR-Gamma), Nuclear factor (erythroid-derived 2)-like 2/antioxidant responsive element (ARE), heat shock factor response element (HSE), mitochondrial membrane potential (MMP), phosphoprotein tumor suppressor (p53), NR (Nuclear receptor signalling pathways), SR (Stress response pathways).

*Confidence score: 0.50-0.69 (low), 0.70-0.80 (medium), 0.80-1.00 (high)

Table _S8: Structural analysis results by DSSP, Naccess, and Dynamut for IndiGen Structure Data.

PDB code	Variation	Sec. Str. Assignment (DSSP)	Solv. Acess. (Naccess)	ΔΔG(Dynamut) kcal/mol	ΔΔSVib	Consurf Score
3D2R	M134V	α-helix	3	-0.595	0.670	-0.696
3GC9	R275H	Turn	76.6	0.156	0.006	1.491
3NRU	I138V	Loop	13.3	0.609	-0.842	1.142
3NYO	V334M	β-sheet	28.6	0.315	-0.057	-0.278
4EYJ	A300T	α-helix	40.8	0.029	-0.128	1.373
4TNB	Q41L	3-10 helix	47.6	-0.205	269	'5
4YHJ	F110V	α-helix	14.5	-1.024	0.844	0.512
4YHJ	Y292A	α-helix	0	-0.041	1.079	-1.037
4YHJ	V247I	β-bridge	1.8	0.379	-0.181	-0.851
4YHJ	C215I	β-sheet	0	0.282	-0.752	-0.83
4YHJ	A142V	α-helix	18.4	0.669	-0.427	1.061
4YHJ	Y53I	Helix turn	6.5	-0.605	0.915	-0.636
4YHJ	R65L	α-helix	43.6	-0.355	0.145	0.863
4YHJ	I33L	Loop	49.8	0.640	-0.133	-0.156
4YHJ	V486A	Turn	76.7	-0.428	0.194	-0.411
4YHJ	F454A	α-helix	14.4	-2.767	1.178	-0.822
5TQY	V155A	Loop	52.1	-0.622	0.073	1.941

6BDN	S47N	Loop	74.9	0.406	-0.065	0.912
6BFN	L521H	Bend	17.3	-0.498	0.140	-0.457
6BFN	F196S	α-helix	74.9	-0.011	0.324	-0.053
6GQ7	T857A	α-helix	70.2	0.080	0.123	0.761
6I83	R982C	α-helix	40.1	0.218	0.129	1.184

Table_S9: PharmaGKB results

Native	Variation	Gene	variants	Chemicals	Phenotypes
4YHJ	F110V	GRK4	rs1024323	metoprolol	Hypertension, Kidney Diseases, Nephrosclerosis
4YHJ	A142V	GRK4	rs1024323	metoprolol	Hypertension, Kidney Diseases, Nephrosclerosis
4YHJ	Y292A	GRK4	rs1801058	metoprolol	hypertensive nephrosclerosis
4YHJ	V486A	GRK4	rs1801058	metoprolol	hypertensive nephrosclerosis
4YHJ	F454A	GRK4	rs1801058	metoprolol	hypertensive nephrosclerosis

Table _S10: Kinase families associated with 327 kinase coding genes with number of drugs and SNPs observed in each class (Indigen Sequence data).

Kinase family	Drug count	SNP count
ACG	339	2343
ATYPICAL	148	1224
CAMK	213	10581
CK1	18	275
CMGC	659	1632
STE	147	1734
TK	1978	5073
TKL	185	1193
OTHER	196	4043

Table _S11: Data used for amino-acid exchange frequency analysis

(Excel sheet- Table_S11.csv)

Table_S12: HUMSAVAR variant data used for comparative structural analysis consisting of 217 variants corresponding to 12 genes in IndiGen structure data.

(Excel sheet- Table_S12.xlsx)

Table _S13: ADR Profile of Atenolol and Metaprolol

(Excel sheet- Table_S13.xlsx)

Table _S14: ADRs and drugs information on the drugs included in our analysis

(Excel sheet- Table_S14.xlsx)

Python script to generate the matrix : https://github.com/raylab-projects/Pharmacogenomics/blob/main/counting_aa_conversion.ipynb
